# Organ damage mitigation with the Baskent Sickle Cell Medical Care Development Program (BASCARE)

**DOI:** 10.1097/MD.0000000000009844

**Published:** 2018-02-09

**Authors:** Hakan Ozdogu, Can Boga, Suheyl Asma, Ilknur Kozanoglu, Cigdem Gereklioglu, Mahmut Yeral, Nurhilal Turgut Buyukkurt, Soner Solmaz, Aslı Korur, Pelin Aytan, Erkan Maytalman, Mutlu Kasar

**Affiliations:** a Adana Adult Bone Marrow Transplantation Center, University Hospital of Baskent, Adana; b Department of Hematology; c Department of Family Medicine; d Department of Physiology; e Department of Immunology, Faculty of Medicine, University of Baskent, Ankara, Turkey.

**Keywords:** chelation, erythrocyte exchange, hematopoietic stem cell transplantation, hemoglobinopathy, mortality, sickle cell disease

## Abstract

The Eastern Mediterranean is among the regions where sickle cell disease (SCD) is common. The morbidity and mortality of this disease can be postponed to adulthood through therapies implemented in childhood. The present study focuses on the organ damage-reducing effects of the Baskent Sickle Cell Medical Care Development Program (BASCARE), which was developed by a team who lives in this region and has approximately 25 years of experience. The deliverables of the program included the development of an electronic health recording system (PRANA) and electronic vaccination system; the use of low citrate infusion in routine prophylactic automatic erythrocyte exchange (ARCE) programs including pregnant women; the use of leukocyte-filtered and irradiated blood for transfusion; the use of magnetic resonance imaging methods (T2^∗^) for the management of transfusion-related hemosiderosis; and the implementation of an allogeneic hematopoietic stem cell transplantation protocol for adult patients. The sample was composed of 376 study subjects and 249 control subjects. The hospital's Data Management System and the central population operating system were used for data collection. BASCARE enabled better analysis and interpretation of complication and mortality data. Vaccination rates against influenza and pneumococcal disease improved (21.5% vs 50.8% and 21.5% vs 49.2%, respectively). Effective and safe ARCE with low citrate infusion were maintained in 352 subjects (1003 procedures). Maternal and fetal mortality was prevented in 35 consecutive pregnant patients with ARCE. Chelating therapy rates reduced from 6.7% to 5%. Successful outcomes could be obtained in all 13 adult patients who underwent allogeneic peripheral stem cell transplantation from a fully matched, related donor. No patients died by day 100 or after the first year. Cure could be achieved without graft loss, grades III to IV acute graft versus host disease, extensive chronic graft versus host disease, or other major complications. The BASCARE program significantly improved patient care and thereby prolonged the life span of SCD patients (42 ± 13 years vs 29 ± 7 years, *P* < .001). We may recommend using such individualized programs in centers that provide health care for patients with SCD, in accordance with holistic approach due to the benign nature but malignant course of the disease.

## 1 Introduction

Sickle cell disease (SCD) is a single-gene disease responsible for the production of abnormal hemoglobin S, which has a tendency to crystallize.^[1]^ The disease is common worldwide. Hemoglobin (Hgb) S incidence was reported to be 10% in the Eastern Mediterranean, namely, in Turkey.^[2,3]^ Painful crises, hemolytic anemia, and organ damage associated with this condition lead to morbidity and mortality.^[3,4]^ Hemoglobinopathy centers, state hospitals, and university hospitals established in the regions where this disease is prevalent are responsible for detection of the disease and the arrangement of educational activities, as well as building awareness and delivering therapies (mainly transfusion therapies, in accordance with current guidelines).^[5]^

Hydroxyurea treatment and improved medical care reduce mortality in childhood; however, organ damage and mortality are merely postponed to adulthood.^[6–8]^ It is clear that interventions are needed to reduce mortality and morbidity in adults. To the best of our knowledge, a limited number of data systems for assessing organ damage, determining patient workflow according to management plans, and ensuring data reliability are available.^[6,9]^ Transfusion policies, which can reduce maternal and fetal losses, may vary among centers.^[8,10]^ Allo-immunization continues to be problematic, despite the development of methods to detect erythrocyte antigens.^[11–13]^ While ferritin levels and trends are widely used for reflecting iron overload, this may not be a reliable marker for SCD patients.^[14]^ Therefore, it is challenging to standardize the chelating therapy and follow-up procedures. Challenges persist in allogeneic transplantation protocols in adults, particularly engraftment problems and graft loss risk.^[15]^ Performing the holistic approach through implementation of a program which facilitates patient follow-up and thereby improves patient care may reduce morbidity and mortality.

The present study evaluated data from the Baskent Medical Care Development Program for Sickle Cell Disease (BASCARE), which was developed by an integrated medical team who have been working in the Eastern Mediterranean for the past 25 years to overcome the challenges of managing SCD.

## 2 Materials and methods

### 2.1 Study plan

This work was designed as a single center, and retrospective chart review including the data between January 2004 and January 2017. A total of 376 patients (155 females) between 17 and 52 years of age who were born in the Eastern Mediterranean consisted of PRANA group. Control group was composed of 249 patients who were being treated by the center but who did not meet regular follow-up criteria. All patients were of Eti–Turk origin and had been diagnosed with homozygous Hgb S or heterozygous combinations of Hgb S and thalassemia (Hgb S-β thalassemia and Hgb S-α thalassemia) by hemoglobin electrophoresis or genetic analysis, and had been regular clients. Patients with Hgb S-α thalassemia may present with as severe as clinical manifestations with homozygous patients probably resulting from racial characteristics. Thus, 2 patients presenting with severe painful crisis and hemolysis in pregnancy were not excluded.

The BASCARE program was developed by four hematologists who had been working at our hospital for at least 10 years (HO, CB, MY, MK), one physiologist (IK), and one family physician (SA), with the aim of improving medical care for SCD patients. The deliverables of the program and dates of onset were as follows: development of electronic recording system in accordance with international accreditation rules (PRANA) and electronic vaccination schedule (beginning from 2012); use of low citrate for automated erythrocyte exchange (ARCE) procedures (2004); implementation of routine prophylactic exchange program for pregnant patients (2008); use of leukocyte-filtered and irradiated blood for blood transfusion (2006); use of magnetic resonance imaging methods for management of transfusion-related hemosiderosis (T2-weighted) (2008), and development of an allogeneic stem cell transplantation (ASCT) regimen for adult patients (2014).

**Figure 1:**
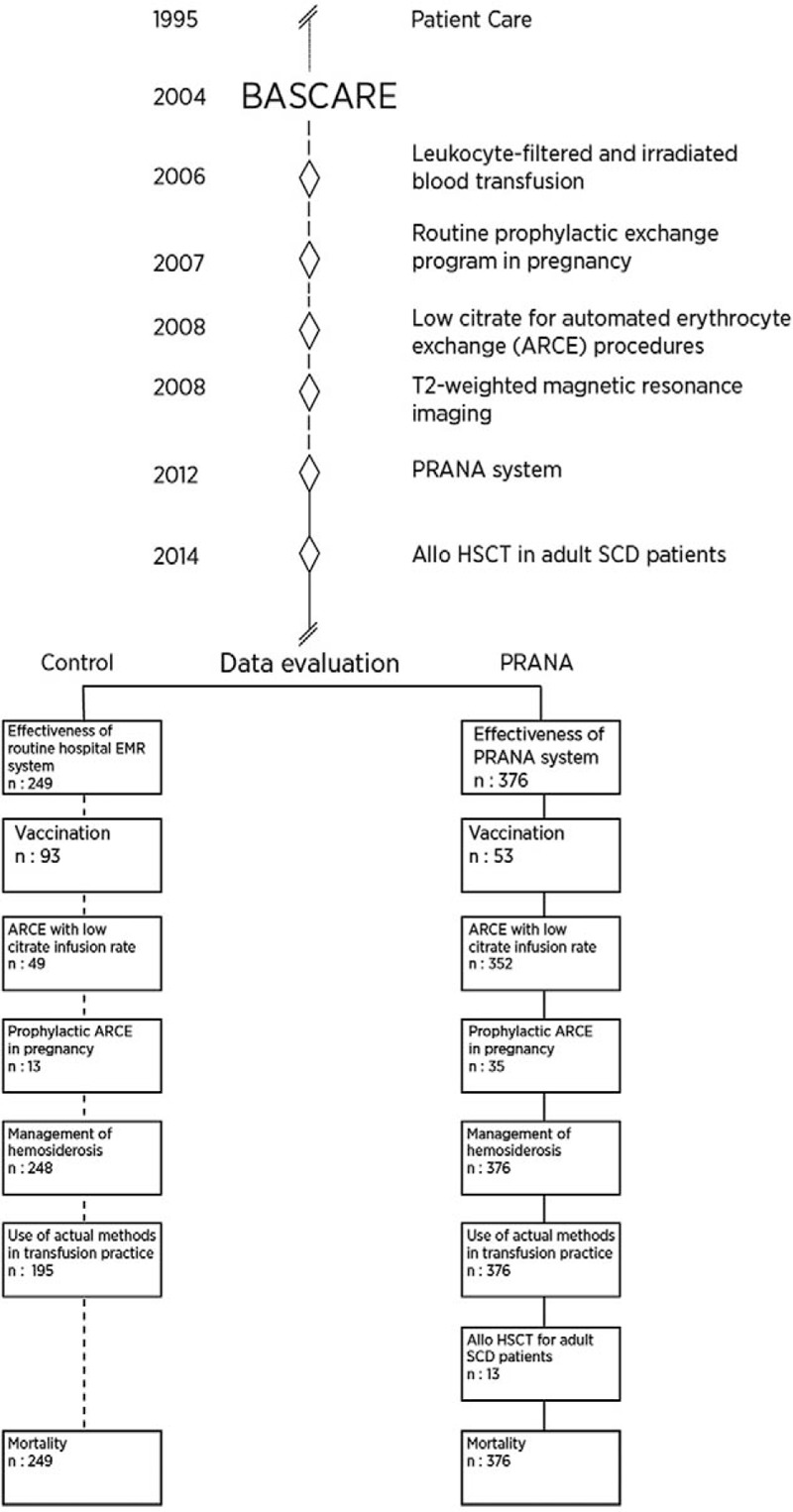
Chronological development of PRANA and data assessment. PRANA yields specific and standard data for sickle cell disease obtained during routine daily clinical practice. Whereas data for control group are collected from routine hospital electronic medical record system contains medical and clinical data gathered in provider offices.

Comparisons between subgroups of PRANA group and control group are presented in Figure 1.

For subgroup analyses, the effectiveness of PRANA system was investigated through checking data of 249 out of 376 patients and the results were compared with those of 196 patients who had been followed-up at the center before the implementation of PRANA system. Ninety-three patients whose electronic vaccination schedule could be reached were used to evaluate the effectiveness of the system. As expected, use of low citrate for ARCE procedures was evaluated in a total of 352 patients including both study and control subjects, only observational data are presented due to missing data before 2004. The procedure was evaluated in 35 pregnant women who underwent prophylactic exchange and in 13 control subjects. The effectiveness of transfusion policy and chelating therapy were investigated by comparing data before and after commencement of the procedures. Observational outcomes of 13 adult SCD patients who underwent ASCT are reported. As a consequence of these improvement efforts, overall mortality age was compared between 376 study and 249 control subjects.

The primary end-point of our study was comparing age at death between the patients who were enrolled and not enrolled in the BASCARE program. The secondary end-points were the time to reach the system to obtain required data and the rates of use for international side effect and complication terminology supported by objective evidence; vaccination rates; clinical effectiveness and side effects of the ARCE with low citrate infusion rate; pregnancy-induced maternal and fetal mortality and morbidity; allo-immunization and transfusion-related graft versus host disease (GvHD) rates; 100-day and one-year mortality rates; and grades I–II and grades III–IV GvHD rates among patients who underwent HSCT.

All patients give consent for use of clinical and laboratory data unless personal identity is not reported. Therefore, no additional written or verbal consent was required from the relatives of the dead patients as this is a retrospective chart review. Local Ethics Committee approval was obtained prior to study commencement (KA17/22).

### 2.2 Data collection and reliability

Data from controls with SCD were extracted from a hospital electronic medical system working through a database system (AviCenna HBYS software version 1.95; Datasel, Ankara, Turkey). Headings included ICD code of diagnosis, symptoms on presentation, history, physical examination, hematologic and biochemical tests, radiology–endoscopy–pathology, consultations, etiology, and treatment and progress. On the other hand, the clinical and laboratory data of the PRANA group patients were obtained from the modular system (PRANA) which has the properties of a electronic health recording system developed by Baskent team in cooperation with engineers (Nucleus, version 9.3.39; Monad Software Company, Ankara, Turkey). Superiorities of PRANA include providing standard data, rapid health information, standardized patient care, standardized workflow in accordance with quality management, and traceability. The Ministry of Health's Central Population Management System was used to confirm mortality records. Age at death was recorded. The data were checked by the JACIE Quality Management Data Audit Group.

### 2.3 Definitions

The patients who come for minimum 2 controls per year during subsequent 3 years were defined as “regular follow-up” patients. Patients who had not required medication to treat painful conditions for the previous 4 weeks were considered to have “steady-state” disease. A “painful crisis” was defined as the need for hospital admission due to pain not related to any cause other than SCD or for the use of parenteral nonsteroidal anti-inflammatory inhibitors, metamizol, and narcotics for the treatment of SCD-related pain. “Nephropathy” was defined as the presence of at least one indicator of renal dysfunction, such as microalbuminuria (30–300 mg of microalbumin per day in spot urine) and proteinuria, hyper-echogenicity and/or thinning of the renal cortex on ultrasonography, and low creatinine clearance. “Pulmonary hypertension” was indirectly determined as a tricuspid jet velocity >2.5 mm/s. “Hydroxyurea use” was defined as the regular use of at least 15 mg of hydroxyurea/kg/day for at least 1 month. “Success after ARCE” was defined as Glasgow–Pittsburgh Cerebral Performance category 1 or 2 for neurologic events, improvement of renal function and liver function (to achieve a postapheresis creatinine and bilirubin value identical to the steady-state value), or the normalization of arterial blood pressure and partial oxygen pressure values.^[16]^ Pain relief and staying in steady state for 1 week was taken as success criteria for prolonged painful crisis. Clinical improvement was the success criteria for priapism, lung damage, and leg ulcer.

### 2.4 Development and implementation of PRANA program

**Figure 2:**
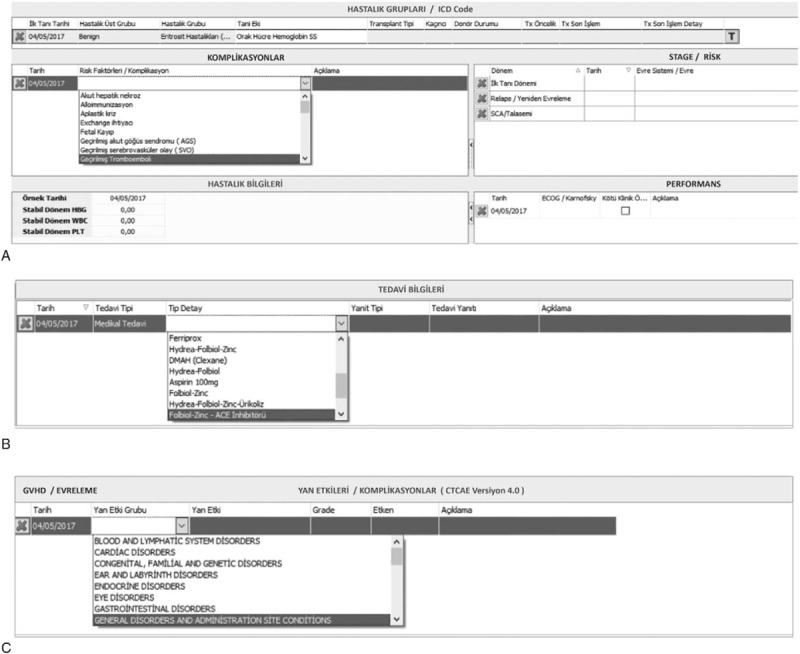
Some parts of PRANA system which is a modular program that records data from SCD patients in the Hospital Data Management Systems. The interfaces which shows (A) disease groups, diagnoses, risk factors, disease stages, and performance status. (B) Treatment data. (C) Side effects and complications according to CTCAE v 4.0.

PRANA is a modular program that records data from SCD patients in the Hospital Data Management Systems so that it can be accessed when needed. Data were stored in the Oracle Database 11g Enterprise ed. 11.2.0.4.064 bit database. This program was developed not only as an electronic medical record system, but also to enable patient follow-up and the monitoring of workflows; it captures complications, patient/donor preparation stages, and the follow-up for patients and donors during and after PSCT. PRANA provides rapid health information. It was composed mainly of a patient file and a coordinator file. The most important parts of the program included ICD codes and risk factors (e.g., acute hepatic necrosis, acute chest syndrome, thrombo-embolism, allo-immunization, number of mean yearly painful crises, nephropathy, osteo-necrosis, pre-eclampsia, and fetal loss), and allowed users to write record details about complications on the same line (e.g., how pulmonary hypertension was evaluated and measurement results). Performance data, steady-state hemoglobin, leukocyte, platelet counts, management plan, response to therapy, side effects, and complications were also recorded according to general terminology criteria (CTCAE v 4.0) (Fig. 2.). The coordinator file included fields for information about council decision, stages of pretransplant preparation for the patient and the donor, and serology of the donor/patient, with automatically recorded consultation and patient/donor follow-up notes.

### 2.5 ARCE with reduced citrate infusion rate

Two different continuous-flow apheresis systems (Cobe Spectra Version 7.0 and Spectra Optia Version 7 until 2015; Terumo BCT, Lakewood, CO, since 2015) were used to exchange 60% to 70% of the patients’ red blood cells with cross-matched donor cells of the same blood type to reduce the Hgb S level to < 30% of the total Hgb content. Until 2008, the inlet pump flow rate and centrifugation speed were automatically adjusted according to preset parameters determined by each patient's data (weight, height, sex, and hematocrit level) during ARCE, during which cells were subjected to 930*g* at a maximum centrifugation speed of 1800 rev min^−1^ (Cobe Spectra Apheresis System Essential Guide). In some patients, the inlet pump flow rate was recorded manually. A range of 15 to 60 mL min^−1^ was used for the flow rate, and the centrifuge speed was established at 400 to 1800 rev min^−1^ (up to 930*g*). The total blood volume was calculated according to the patient's body weight (70–75 mL kg^−1^), and the calculation of red cell volume was based on hematocrit values.^[2]^ Acid–citrate–dextrose A solution (ACD-A) (whole blood to ACD-A ratio = 15:1) was used in all procedures. A device developed for ARCE (Spectra Optia) was implemented after 2012, although the procedure was performed using a low citrate infusion rate. This device objectively showed the amount of citrate (mL) given to the patient, which had not been shown by the Cobe Spectra. The novel device also performed the rinse procedure automatically, instead of manually.

The target Hct level was guided by the occurrence of a complicated pain episode and/or by the steady-state hematocrit level noted in SCD patients who were regularly examined as outpatients.^[16,17]^ The patients who experienced a complicated pain episode required hyper-transfusion, and a target Hct value of 30% to 34% after the exchange was arranged to ensure a positive outcome. Our goal for the remaining patients was to achieve a final post-exchange Hct value identical to the steady-state value.

### 2.6 Routine prophylactic ARCE in pregnancy

The prophylactic ARCE treatment was administered in the second and third trimesters. As the patients undergoing exchange transfusion required negative direct and indirect antiglobulin tests, these tests were performed before the ARCE procedure in all patients. The target Hct levels were determined according to the steady-state Hct levels of patients who were regularly examined as outpatients. The need for RBC transfusion was averaged according to the target Hct level. Patients who had a complicated pain episode, characterized by clinical deterioration, a Hgb level of < 7 g/dL, leukocytosis in the absence of infection, and an underlying pulmonary or cardiac disease required hyper-transfusion with a target Hct of 27% to 28% after the exchange.^[17]^

### 2.7 Management of hemosiderosis

All imaging analyses were performed as previously described with slight modifications, and a 1.5T MRI system was used for these analyses (Avanto; Siemens, Erlangen, Germany). Briefly, liver and myocardial measurements included T2^∗^ value screenings. The signal intensity of these regions for each echo time was measured and plotted as an exponential signal decay curve. The lower limit of normal for T2^∗^ in the detection of myocardial iron deposition has been reported to be 20 ms, and this value was used as the cut-off in this study. A T2^∗^ value of >20 ms indicated no cardiac iron overload, while ≤20 ms indicated that such overload had occurred. Liver iron deposition was evaluated using the R2^∗^ value (R2^∗^=1000/T2^∗^). The R2^∗^ value was converted to a liver biopsy equation using the calibration curve drawn during the study.

MRI T2^∗^ measurements were carried out for patients whose serum ferritin level was 1000 μg/L or above and who regularly receive blood transfusion. A liver iron concentration in dry tissue of >3.2 mg Fe/g was regarded as hepatic siderosis.^[18–21]^ Deferasirox treatment was started for patients who were found to have tissue iron deposition (Exjade, Novartis, Switzerland). The initial daily dose was 20 mg/kg, per os. All patients required escalation of 5 to 10 mg/kg per daily dose to enable serum ferritin to fall below the baseline. Decision for stopping chelating therapy was supported by radiological evidence.^[14]^

### 2.8 Use of actual methods in transfusion practice

Leuko-filtered red cell suspensions preserved for 1 to 7 days by citrate–phosphate–dextrose and with an Hct level of about 75% were used. The RBCs were matched for Rh (C, c D, e) and Kell (K) blood group antigens to minimize allo-immunization. Simple erythrocyte transfusion and some packaged erythrocytes used for ARCE could be obtained from the close relatives of the patients. Therefore, erythrocyte bags were irradiated using a dose of 25–50 cGy with an irradiation device that uses cesium 137. The patients who attended and did not attend regular controls were compared with regard to allo-immunization and transfusion-related GvHD.

### 2.9 Hematopoietic stem cell transplantation

The adult patients who had an indication for transplantation underwent ASCT from an HLA-matched sibling donor using peripheral blood. Indications for transplant included one or more of: frequent painful crises despite hydroxyurea treatment (>2/year), pulmonary hypertension (TRV >2.5 m/s), acute neurological attack, acute chest syndrome, silent cranial ischemia, widespread osteo-necrosis, and allo-immunization. A protocol that facilitated engraftment, hindered graft loss, and minimized acute and chronic GvHD was developed owing to engraftment and graft loss problems seen in transplants performed with nonmyeloablative regimens. The conditioning regimen included 200 cGy TBI and Flu150/Bu 3.2/Cy29/ATG-F 30. GvHD prophylaxis was performed with sirolimus, mycophenolate mofetil, and post-transplant Cy (100/kg).^[15]^

### 2.10 Statistical methods

Statistical analysis was done using SPSS 17.0 (SPSS Inc., Chicago, IL). Categorical measurements are expressed as the number and percent, and continuous variables as the mean ± standard deviation or median and minimum–maximum, when required. Categorical variables were compared using the χ^2^ test or Fisher's exact test. Distributions were controlled in comparisons of mortality and continuous measurements; Student's *t*-test and the Mann–Whitney *U* test were used in analyses of parametric and nonparametric variables, respectively. A *P* value of < .05 was considered to indicate statistical significance.

## 3 Results

### 3.1 Effectiveness of PRANA system

The BASCARE application facilitated the collection of clinical data about SCD. When the routine hospital's Data Management System and electronic health recording system PRANA were compared, the time taken to access the required data was 84.9 ± 39.0 s versus 3.36 ± 0.70 s for steady-state hgb; 100 ± 51 s versus 2.8 ± 0.4 s for the rate of painful crisis per year; 86 ± 53 s versus 4 ± 1 s for pulmonary hypertension; 117 ± 67 s versus 3.4 ± 1.0 s for bone necrosis; and 95 ± 57 s versus 3.8 ± 1.0 s for proteinuria (*P* = .01 for all). The proportion of missing data decreased with PRANA. For example, the proportion of missing data for steady-state Hgb decreased to 18% from 42% (*P* = .001). This application increased the use of common terminology criteria for adverse events supported by objective evidence to 100% (system included CTCAE vs 4.0 and mandated data entry). This possibility did not exist before PRANA.

Comparison of groups with regard to acute and chronic organ damages is presented in Table 1. Data of only 49 patients could be reached in control group. Organ damages seem to be better controlled in PRANA group (Table 1).

**Table 1:**
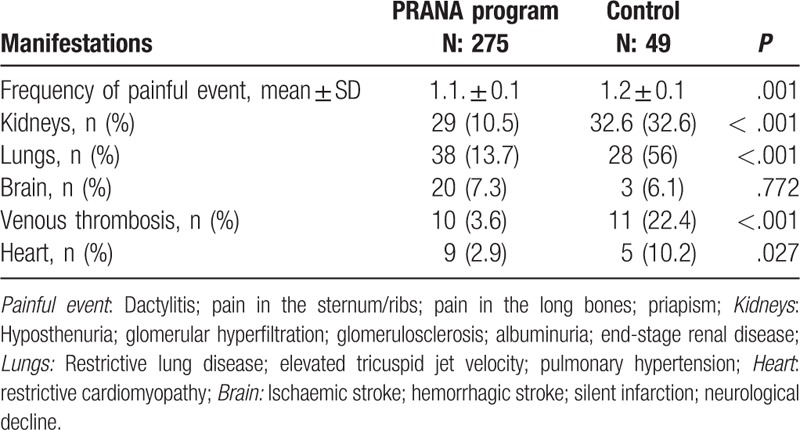
Comparison of acute and chronic organ complications of sickle cell disease by groups.

### 3.2 Electronic vaccination system

The electronic vaccination system menu consisted of vaccines against influenza, pneumococcal disease, hemophilus influenza type b, meningococcal disease, and hepatitis B, according to the recommendations of Infectious Diseases Society of America. HBsAg positivity was detected in 2 out of 93 cases (2.1%), anti-HBsAg positivity was detected in 7 (7.5%), and anti-HCV positivity in 6 (6.4%) in 2011, before this system was introduced. Twenty patients (21.5%) were found to have been vaccinated against pneumococcal disease and influenza. No patients had been vaccinated against *Haemophilus influenzae*.

After the system had been introduced, 40 (43%) patients were vaccinated against hepatitis B. Antibody titers against hepatitis B became positive in 47 (50.5%) patients (>10 IU/mL). When 53 patients who came for regular checks were analyzed, the numbers with vaccination against pneumococcal disease and influenza were found to be 29 (49.2%) and 30 (50.8%), respectively (*P* < .005). Therefore, the electronic vaccination system that notified the physician about the absence of vaccination was effective for improving vaccination rates.

### 3.3 ARCE with reduced citrate infusion rate

In PRANA group, a total of 352 subjects (169 F, 183 M), ranging from 15 to 58 years of age, underwent prophylactic erythrocyte exchange with reduced citrate infusion rate. A total of 1003 procedures were performed. Indications for the procedure and clinical success rate are shown in Table 2. Prolonged painful crisis was the most common indication for ARCE and a statistically significant difference was found between groups with regard to success rate in favor of PRANA group (98.7% vs 91.0%; *P* = .02). Success rate was higher in PRANA group indications like lung damage, acute neurologic event, and leg ulcers (*P* = .008, *P* = .010, and, *P* = .001, respectively).

**Table 2:**
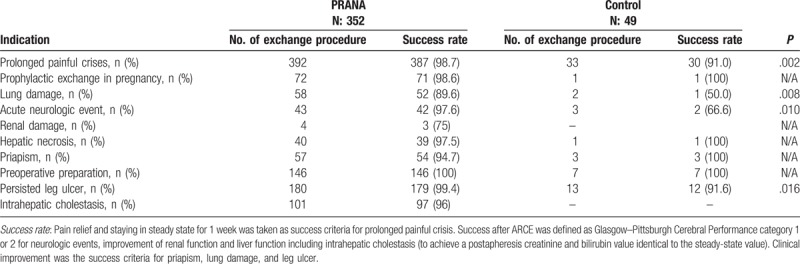
Indications for erythrocyte exchange and comparison of success rates by groups.

Procedural side effects were rare and could be easily managed (vascular access problems occurred in 10 cases [1%], vasovagal syncope in 1 [0.09%], paresthesia in 2 [1.9%], and allergic reaction in 15 [1.4%]). No patient exhibited anaphylaxis, hypocalcemia, or neurological symptoms (e.g., apathy, convulsion, headache).

### 3.4 Prophylactic ARCE in pregnancy

A total of 35 patients aged between 20 and 43 years of age underwent 73 prophylactic ARCE. Nineteen patients (54.2%) underwent 2 procedures, one in the second and one in the third trimester. Maternal or fetal loss did not occur. HELLP syndrome developed in 1 patient (2.3%) and vaginal hemorrhage developed after cesarean section in 2 (5.7%) patients. IAT tests were positive in 4 (11.4%) cases. Vaso-occlusive crises developed in 5 out of 13 subjects (38.5%) who did not undergo prophylactic ARCE during pregnancy and 4 (30.7%) died. Fetal loss developed in one (7.7%) subject and hypertension in one (7.7%) subject.

### 3.5 Management of hemosiderosis

For hemosiderosis management, 134 radiological analyses were done in 119 (32%) patients in PRANA group and 77 analyses in 53 (21%) controls (*P* = .004). As the results of these analyses, cardiac hemosiderosis was detected in 2 (1.6%) patients in PRANA group and in 1 (1.8%) controls (*P* = .920). These numbers were 19 (14%) versus 7 (9%), respectively, in PRANA and control groups for hepatic hemosiderosis (*P* = .640).

While 13 out of 195 (6.7%) patients in control group were detected to receive iron-chelating therapy, this ratio was 19/376 (5%) in PRANA group (*P* = .420). These numbers represent the patients who had started chelating therapy during the study period and also the ones who had been already using therapy. When these patients were evaluated using MRI T2^∗^ measurements, 5 (1.3%) patients in PRANA group could discontinue chelating therapy, no data could be reached for controls as the patients were lost to follow up.

### 3.6 Use of actual methods in transfusion practice

Indirect antiglobulin test was applied to 240 out of 376 patients and 30% were detected to be allo-immunized despite preparing erythrocyte components, and using leukocyte-filtered and irradiated blood. Transfusion-related GvHD did not develop in any cases, despite using blood obtained from close relatives because of socio-cultural factors.

### 3.7 Hematopoietic SCT for adult sickle cell disease

A total of 13 adult patients (5 females and 8 males) between 20 and 46 years of age underwent peripheral SCT from HLA full-matched relatives through the BASCARE protocol. The mean follow-up period was 15 months (range 6–33). Transplant-related mortality and 1-year mortality were both zero. In the early period, 1 patient (7%) developed easily managed grade II acute GvHD and infectious complications (CMV antigenemia in 4 cases [30%] cases, bacterial infection in 3 [23%], and BKV infection in 6 [46%]). Significant late complications did not develop in any cases (unpublished data).

Three out of 13 patients has pulmonary hypertension (TRV≥ 2.5) before transplant, however, control tests could not be done as three patients did not complete 1 year after transplant.

### 3.8 BASCARE and mortality

**Figure 3:**
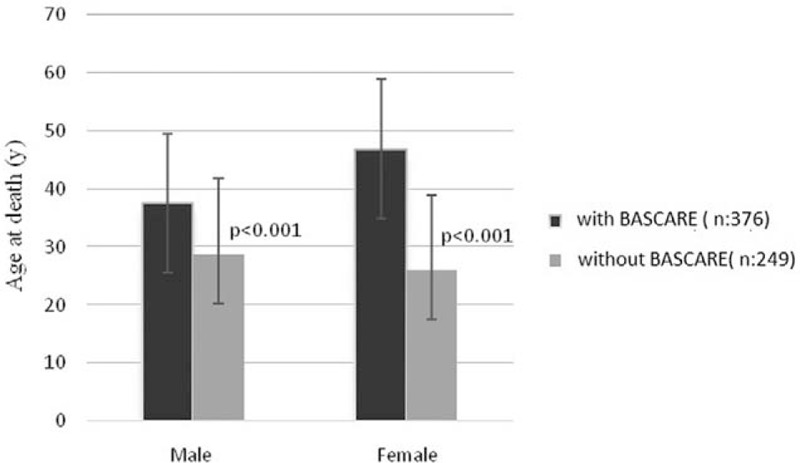
Comparison of age at death in the patients who were followed-up with or without BASCARE program. BASCARE = Baskent Sickle Cell Medical Care Development Program.

When the influence of the BASCARE program on crude mortality rate was evaluated in adult SCD patients, 27 out of 376 patients who were enrolled in the PRANA system were found to have died (7.1%). Twenty-one out of 249 patients who were not included in this system (8.4%) died within the same period. The mean age at death was 42 ± 13 years (for males 21–59 years, mean age 38 ± 13 years; for females 27–61 years, mean age 47 ± 13 years) in the group included in the study. On the other han6d, the mean age at death was lower (29 ± 7 years) in the group that was not included in the program (for males 27–40 years, mean age 32 ± 6 years; for females 17–35 years; mean age 26 ± 9 years). There was a statistically significant difference in age at death between the groups with regard to age at death (*P* < .001) (Fig. 3.).

When the groups were compared with regard to causes of death, while the most common cause was found as acute chest syndrome in 9 patients (33.3%) in PRANA group, this number was 2 (9.5%) in control group but the difference was not statistically significant. Number of the cases whose cause of death could be identified was higher in PRANA group compare to control group (18.5% vs 61.9%; *P* < .001) (Table 3).

**Table 3:**
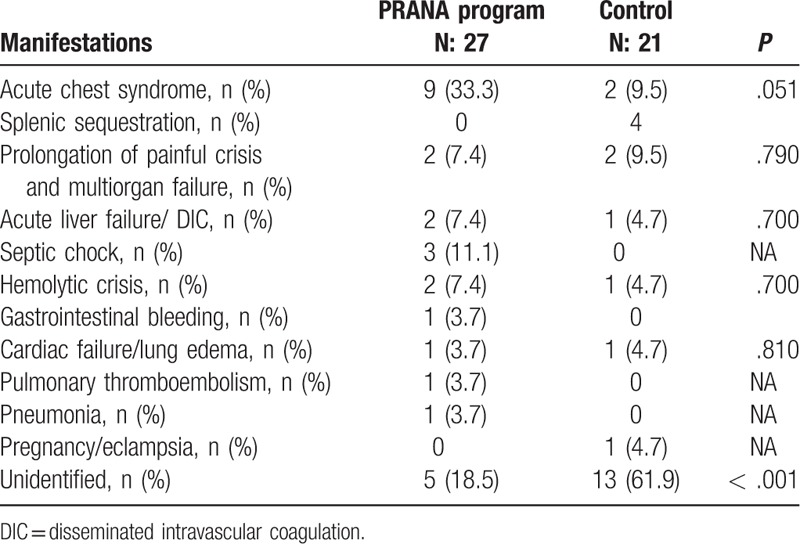
Causes of death by groups.

## 4 Discussion

The clinical course of SCD is similar to that of malignant diseases, although it is a nonmalignant disease. Microischemia and inflammation are responsible for progressive and severe organ damage.^[7,8]^ The quality of life is severely impaired for patients, owing to the occurrence of painful crises and medical problems.^[3,7,8]^ The mean age of mortality for the disease is 39 years.^[1]^ A multicenter mortality study conducted in the Eastern Mediterranean found that crude mortality was 40.1 ± 15 (17–64) years for females and 34.1 ± 10 (18–54) years for males. Acute chest syndrome, splenic sequestration, and prolonged painful crisis-related multiorgan failure were the most common causes of mortality. Morbidity and mortality rates varied among ethnicities and the countries where the patients lived.^[22]^ While a significant difference could not be found between groups with regard to causes of mortality, higher number of patients with identified causes in PRANA group may be associated with regular patient records.

Unfortunately, the vast majority of the patients cannot benefit from current medical developments for the following reasons. Few centers have experience with SCD patients, and patient follow-ups may be insufficient in the centers where the disease is not prevalent.^[23–25]^ More importantly, SCD is a special disease and its management requires a well-structured multidisciplinary approach. Therefore, the absence of a special organization and integrated team, as well as insufficient blood bank services, may impair patient care. The attending physicians should play a role in correctly informing hospital management. Patient-related factors are also important, such as patient adherence to hydroxyurea treatment.^[26–28]^ Economic factors and emotional problems may also make patient compliance challenging.

Laws and regulations force institutions to improve medical care for SCD patients. The individual efforts of medical staff who have worked with SCD patients for several years are focused mainly on hydroxyurea treatment and the development of transfusion policies. The main output of these efforts is the guidelines developed for SCD management.^[29,30]^ However, the centers are not objectively evaluated for their adherence to these guidelines because SCD is a heterogeneous disease and requires individual-specific treatments.

The most important deficiency in the management of these patients is the absence of a standard workflow. Although such a workflow may be present, its implementation is usually inadequate. On the other hand, developments in the medical field may not be reflected in daily practice owing to obstacles of knowledge/experience, as well as financial and technical problems like novel blood bank methods, apheresis, transfusion, and stem cell transplantation techniques. An electronic recording system developed based on patient characteristics determines the workflow, and traceability is the most valuable tool for these patients.^[31]^ Here, we explored the development of a electronic health recording system (PRANA) that meets international accreditation rules (authorization, coding, data safety, storage, traceability, retrospective data collection, warnings, international terminology). This system has become integrated into the daily routine for patient follow-up. The PRANA system also allowed us to implement a multifunctional electronic vaccination system that alerted physicians at the beginning of the examination and prompted them to check the vaccination status of the patients. Despite the improved vaccination rates in PRANA group, this ratio could not reach 100% due to reimbursement and low economic status of the patients.

Starting chelating therapy and disease management is an issue that is frequently discussed. SCD is an inflammatory disease, and elevated serum ferritin levels therefore cannot be a reliable parameter for assessing iron overload.^[18,21]^ Moreover, the outcomes of chelating therapy during pregnancy are obscure, and risks of the drugs cannot be neglected.^[30]^ The need for pretransplant chelating therapy, the duration of therapy, and treatment goals have not yet been fully determined. Imaging techniques to reveal tissue iron levels may be helpful for preventing unnecessary drug use. As expected, MRI T2^∗^ measurements could be performed in higher number of patients in PRANA group due to being under regular control and thereby some part of the patients could discontinue chelating therapy. This ratio could not be obtained for control group as they lost to follow up.

ARCE is associated with 2 major adverse events. The first is citrate-related complications^[16,17]^, and the second is allo-antibody development in the late stages of disease.^[13,17]^ Reduced citrate infusion in the exchange protocol was shown to lead to acceptable side effects in many procedures. Allo-immunization continues to be a problem, despite recent developments in blood bank techniques and transfusion medicine. Our results indicate that subgroup analysis using leukocyte filters may be effective in reducing allo-immunization, although it cannot fully prevent occurrence. It must be remembered that SCD patients tend to receive blood products from close relatives, which can lead to transfusion-related GvHD. The use of irradiated blood may reduce this side effect. Given that all patients in both groups underwent reduced citrate ARCE using the same equipment by the same team, the difference between groups in favor of PRANA group with regard to success rate for painful crisis, acute neurologic event, lung damage, and leg ulcer may suggest the contribution of regular follow up and patient records to improved patient care.

The current literature indicates that prophylactic erythrocyte exchange may be beneficial during pregnancy.^[12,17]^ No maternal and fetal losses were detected in pregnant woman who were being followed-up in the context of the BASCARE program and who underwent prophylactic ARCE. In the control group there were 4 maternal deaths from vasoocclusive crisis. This supports strongly the use of some form of transfusion during the pregnancy.

The cure rate is known to be about 90% in ASCTs from HLA full-matched relative donors to pediatric patients who have not developed SCD-related organ damage. Reduced-intensity conditioning regimens are preferred in adult patients owing to the risk of organ damage. Recent studies have revealed that success rates in adult transplants were close to those in children.^[32–34]^ The main complications in adult transplants include engraftment, early or late graft loss due to inflammation, and hyper-proliferative bone marrow.^[15,34]^ Endothelial activation is also an important characteristic of SCD.^[35]^ Given that the endothelium is the target tissue for allogeneic cells in GvHD, it is obvious that measures to prevent GvHD are of vital importance. The conditioning regimen and GvHD prophylaxis were intended to overcome these 2 major challenges in the context of the BASCARE program, and prevented mortality, grades III–IV GvHD, and widespread cGvHD. The program is still being used successfully. It has enabled the medical team to obtain objective data for transplant indications through regular follow ups and correct patient record. The success of the program may be attributed to the suppression of inflammation before transplantation, the use of a less toxic conditioning regimen, sufficient myelosuppression, and the current post-transplant cyclophosphamide treatment for GvHD prophylaxis.

This study has some limitations. First is the absence of a comparison between low citrate ARCE and control subjects. Second is small number of transplant patients and short duration of follow-up after ASCT. Third is large number of missing data belonging to the former recording system supporting our hypothesis for the need to develop a novel and improved system. Small number of the control group in some of the analyses may also be considered as a limitation.

In conclusion, this study showed that implementation of the BASCARE program significantly improved the age of mortality, the management of SCD, and consequently increased the motivation of the medical team. The use of such programs is strongly recommended in all centers that provide healthcare for SCD, which is a nonmalignant disease with many of the features of a malignant disease and has a mean age of mortality of 36 years.

## 5 Author contributions

CB, SA, and HO conceived and designed the project. CG, PA, MY, PA, SS, AK, NTB, MK, EM, and IK collected and analyzed the data. CB and CG wrote and finalized the paper.

*Compliance with ethical standards*: This study was approved by the local ethics committee at the Baskent University, Ankara, Turkey, in accordance with the ethical standards of the 1964 Declaration of Helsinki and its later amendments.^[36]^

## Acknowledgments

We acknowledge Fatih Kandemir, Basak Aydemir, Berna Kutlu, and Evren Boga for their technical help.
